# Nursing and midwifery students’ experiences and perception of their clinical learning environment in Malawi: a mixed-method study

**DOI:** 10.1186/s12912-020-00480-4

**Published:** 2020-09-14

**Authors:** B. C. Mbakaya, F. W. Kalembo, M. Zgambo, A. Konyani, F. Lungu, B. Tveit, A. Kaasen, M. Simango, T. Bvumbwe

**Affiliations:** 1 Nursing Department, St John’s Institute for Health, Mzuzu, Malawi; 2 Faculty of Health Sciences, Mzuzu University, Mzuzu, Malawi; 3 School of Nursing, Midwifery and Paramedicine, Curtin University, Perth, Australia; 4 School of Nursing and Midwifery, Edith Cowan University, Perth, Australia; 5 Faculty for Health Studies at VID Specialized University, Oslo, Norway; 6 Faculty of Health Sciences, Oslo Metropolitan University, Oslo, Norway; 7 Norwegian Church Aid - Malawi office, Lilongwe, Malawi

**Keywords:** Nursing, Midwifery, Students, Satisfaction, Learning, Environment

## Abstract

**Background:**

The clinical learning environment is an important part of the nursing and midwifery training as it helps students to integrate theory into clinical practice. However, not all clinical learning environments foster positive learning. This study aimed to assess the student nurses and midwives’ experiences and perception of the clinical learning environment in Malawi.

**Methods:**

A concurrent triangulation mixed methods research design was used to collect data from nursing and midwifery students. Quantitative data were collected using a Clinical Learning Environment Inventory, while qualitative data were collected using focus group discussions. The Clinical Learning Environment Inventory has six subscales of satisfaction, involvement, individualisation, innovation, task orientation and personalisation. The focus group interview guide had questions about clinical learning, supervision, assessment, communication and resources. Quantitative data were analysed by independent t-test and multivariate linear regression and qualitative data were thematically analysed.

**Results:**

A total of 126 participants completed the questionnaire and 30 students participated in three focus group discussions. Satisfaction subscale had the highest mean score (M = 26.93, SD = 4.82) while individualisation had the lowest mean score (M = 18.01, SD =3.50). Multiple linear regression analysis showed a statistically significant association between satisfaction with clinical learning environment and personalization (β = 0.50, *p* = < 0.001), and task orientation (β =0.16 *p* = < 0.05). Teaching and learning resources, hostile environment, poor relationship with a qualified staff, absence of clinical supervisors, and lack of resources were some of the challenges faced by students in their clinical learning environment.

**Conclusion:**

Although satisfaction with clinical learning environment subscale had the highest mean score, nursing and midwifery students encountered multifaceted challenges such as lack of resources, poor relationship with staff and a lack of support from clinical teachers that negatively impacted on their clinical learning experiences. Training institutions and hospitals need to work together to find means of addressing the challenges by among others providing resources to students during clinical placement.

## Background

Clinical learning environment plays an important role in influencing students’ learning behaviours and acquisition of nursing and midwifery clinical competencies [1–3]. The clinical learning environment enables students to bridge the theory-practice gap and obtain the critical skills necessary for clinical decision making [3]. Nursing and midwifery students spend more time in clinical settings than in the classroom during their training period to facilitate the acquisition of clinical skills [4]. According to Flott and Linden [5], the clinical learning environment includes four attributes that impact student learning: the physical space, psychosocial and interaction factors, organisational culture, and teaching and learning components.

The physical space encompasses the environment and resources that influence learning [6] including equipment, facilities, learning tools and standard procedures [2]. While teaching hospitals need to have good facilities, equipment, and learning tools to improve the clinical learning experience of nursing and midwifery students, many hospitals in Malawi and other sub-Saharan Africa countries lack such resources [7, 8]. However, inadequate teaching resources in hospitals is also evident in high-income countries. A qualitative study among undergraduate nursing students in Norway identified lack of equipment, and unfamiliar, old and outdated equipment as challenges to the physical learning environment [2]. The scarcity of resources has a negative impact on students’ learning as they are forced to improvise when providing nursing care to patients [2]. The clinical learning environment, therefore, should be well resourced and organised to enhance the acquisition of knowledge and skills.

The psychosocial and interaction factors of the clinical environment encompass communication, behaviours and attitudes displayed by a qualified healthcare worker, clinical instructors and students that influence clinical learning [9]. Students have identified lack of clearly stipulated expectations in the clinical learning environment as one of the significant challenges that are faced during their clinical practicum [2]. Furthermore, the authors of an Iranian study reported that clinical instructors, who were verbally abusive, created a hostile learning environment that demotivated students to perform procedures in the ward [10]. Contrarywise, authors of another Iranian study reported that avoiding yelling or use of harsh words by clinical instructors when communicating with students in the ward, enhanced positive clinical learning experience [9].

Organisation culture is another important component of the clinical learning environment. It is related to the healthcare managers’ perception of nursing education, organisational policies related to students scope of practice and the provision of quality care to patients [9, 11]. Nursing managers have the responsibility to guide and give adequate time to qualified nurses to support students [12]. The nursing managers need to promote a culture of learning and teaching through equipping staff with knowledge and skills to support students, assigning qualified staff to partner with students on a shift, and allocating reasonable workload to qualified nurses to allow time to teach students [12].

The teaching and learning components involve the process and effectiveness of teaching, supervising and evaluating students in the clinical area by their clinical instructors. Literature shows that students acquire clinical competencies most effectively in the clinical environments where they participate in the provision of care and work alongside healthcare staff that support and encourage learning [13, 14]. The process of how the required competencies are acquired needs close monitoring to make sure that the clinical learning program fits the purpose. Nursing students are evaluated in clinical learning environments where skills and knowledge are applied to patient care [5]. Nonetheless, qualitative findings from two studies conducted in South Africa and Tanzania demonstrated that students lacked adequate clinical supervision because clinical facilitators were often not available or were spending less time with them in the clinical area [15, 16].

Like other resource-limited countries, Malawi has a critical shortage of nurses. For example, the current nurses to population ratio is 3.4:10,000, which is a third of the World Health Organisation (WHO) standard recommendation [17]. Nursing and midwifery institutions in Malawi have responded to the critical shortage of nurses by increasing enrolment numbers of students. In addition, teaching institutions in Malawi have integrated nursing and midwifery training courses to meet the demand for nurses and midwives in the country. The integrated program involves students completing both midwifery and nursing units during their training program. However, these strategies are depleting the already limited resources at the teaching hospitals that are allocated with large numbers of students per period for clinical practice. Furthermore, nurses in Malawi report lacking resources, feeling exhausted and failing to support students because of high workloads [17]. Although nurses in Malawi feel less equipped to adequately support students during their clinical practice, little is understood about the experiences and perceptions of student nurses and midwives of their clinical learning environment. Therefore, this study was undertaken to respond to the following specific research questions: 1). What are the nursing students’ experiences and perception of their clinical learning environment; and 2). What are the psychosocial characteristics of the clinical learning environment that are associated with satisfaction with the clinical learning environment?

## Methods

### Study design

This study used a concurrent triangulation mixed-methods design [18]. Quantitative and qualitative data were collected concurrently, analysed separately, and the results were compared. While the quantitative part of the study helped to establish the student nurses’ levels of satisfaction with their clinical environment, the qualitative component provided a rich account of the students’ experiences with their clinical learning environment. The design was chosen to validate the findings of one method with the other as a means of obtaining comprehensive and credible evidence of the research problem. Creswell [19] refers to this validation of findings from the two methods as confirmation or corroboration. The comparison or integration of the findings is normally done in the discussion section of the study [19]. This design is recommended because it is efficient, as qualitative and quantitative data can be collected at the same time [19, 20].

### Study sites, study population and recruitment criteria

Study participants were recruited from three nursing and midwifery training institutions in the Northern part of Malawi. These were the only generic nursing and midwifery training institutions in the region at the time of data collection. Nursing and midwifery students were recruited in the study if they:1) were in year two to four of study; 2) had a minimum of one clinical placement experience, and 3) were from a nursing and midwifery training institution from the Northern Region of Malawi.

### Sampling

Conventional convenient sampling method was used to recruit students to the study [21]. Clinical facilitators distributed research information and consent packages to a total of 133 students who were doing clinical placements in various hospitals in Malawi. Of these, 126 students consented to participate in the survey representing a response rate of 94.7%. Seven students did not return the signed consent forms. Out of the 126 students, 30 students further consented to take part in the focus group discussion.

### Procedure

#### Survey

Ethical approval for the study was obtained from the Malawi National Health Sciences Research Committee (NHSRC). Permission to conduct the study was sought from the managers of the hospitals and the nursing and midwifery training institutions. The clinical facilitators in these hospitals distributed the study information sheet and consent forms to potential participants. The potential participants were given information about the aims, benefits, risks, and procedure of the study. The participants were also assured of their confidentiality, privacy, and their right to withdraw from the study without an impact on their training. Those who returned signed consent forms were given a self-administered questionnaire that took approximately 10 to 15 min to complete. Although researchers were given rooms within the hospitals to use for the project, students completed the questionnaires at their chosen place and time of comfort.

#### Focus group discussions

Thirty of the participants who completed the questionnaire verbally consented to participate in the focus group discussions. Three focus groups discussions (one per training institution) were undertaken. Each focus group discussion comprised of 10 students from the same training institution. Female authors AK and FL, who were nursing lecturers with qualitative research backgrounds, facilitated the focus group discussion. The discussions were conducted in a quiet room within the hospitals’ premise. A focus group discussion guide was used to ensure that the topics around experiences and perception of clinical learning environments were discussed uniformly. The focus group discussions were audio-recorded and both facilitators recorded field notes which, were considered and included in the qualitative analysis. The focus group discussions took between 45 to 80 min to complete.

### Study measures

#### Survey questionnaire

Data were collected using a self-administered questionnaire, which had two sections; sociodemographic characteristics of participants and the clinical learning environment inventory (CLEI) [22]. The sociodemographic characteristics of the questionnaire collected information such as age, year of study, a program of study (diploma/degree), duration and the number of the clinical placements completed since the start of the study program. The second section of the questionnaire had questions from the CLEI. CLEI has two types of assessment dimensions called the ‘the actual form’ and the ‘preferred form’. The Actual Form is used to assess students’ perception of the real clinical learning environment, while the ‘Preferred form’ is used to assess students’ perception of the characteristics of the desired clinical learning environment. The Actual Form was more suitable and therefore, used in this study. The questionnaire comprises of 42 items that are grouped into six subscales of seven items each. The subscales are: 1) Satisfaction- students enjoyment with the clinical placement; 2) Involvement- students involvement in hospital activities; 3) Individualisation- extent to which students are allowed to make decisions in the clinical area; 4) Innovation - clinical teacher innovative teaching strategies; 5) Task orientation - organisation and clarity of ward activities; and 6) Personalisation- students opportunities to interact with clinical teacher and concern for students welfare. The responses to each item are rated on the 4-point Likert scale: 1 = Strongly agree, 2 = Agree, 3 = Disagree, and 4 = Strongly disagree. The scale has been validated and has good reliability (Cronbach’s alpha =0.73–0.84) [22, 23]. The instrument has been previously used in Malawi and other developing countries [24–26]. Higher scores after total summation indicate a high level of satisfaction with the clinical placement.

#### Focus group interview guide

A structured interview guide was used to collect qualitative data during the focus group discussion. The interview guide contained open-ended questions about students learning experiences, expectations, working relationships, teaching methods and challenges faced during the most recent clinical placement (see Appendix 1). The interview guide was developed by the research team guided by the review of the extant literature. Expert's opinion was sought to validate the interview guide.

### Pilot study

The data collection instruments were piloted with 10 nursing students from a nursing institution, which was not part of the our study sites. This was done to ensure that the instruments were applicable to the Malawian socio-cultural setting. The piloted data were analysed and where necessary, changes were made to the data collection tools.

### Data analysis

#### Quantitative data

Descriptive analysis was used to provide the general characteristics of participants. The mean scores of CLEI and its subscales were analysed by independent t-test and ANOVA, while multiple linear regression was conducted to assess the relationship between nursing students’ satisfaction with the clinical learning environment and the psychosocial characteristics of CLEI. *P*-value was significant at 0.05. The Statistical Package for the Social Sciences (SPSS) version 23 was used to analyse the data.

#### Qualitative data

For qualitative data, all the focus group discussion were conducted in English because it is a learning medium for nursing training institutions in Malawi [27]. All the focus group discussions were audio-recorded, transcribed and member checked. Field notes were taken immediately following each focus group discussion. Data were analysed manually guided by Braun and Clark’s six phases of thematic analysis [28]. The six phases are data familiarisation, generation of initial codes and collating data according to the codes, searching for themes, reviewing themes, defining and naming themes, and producing a report.

**Fig. 1:**
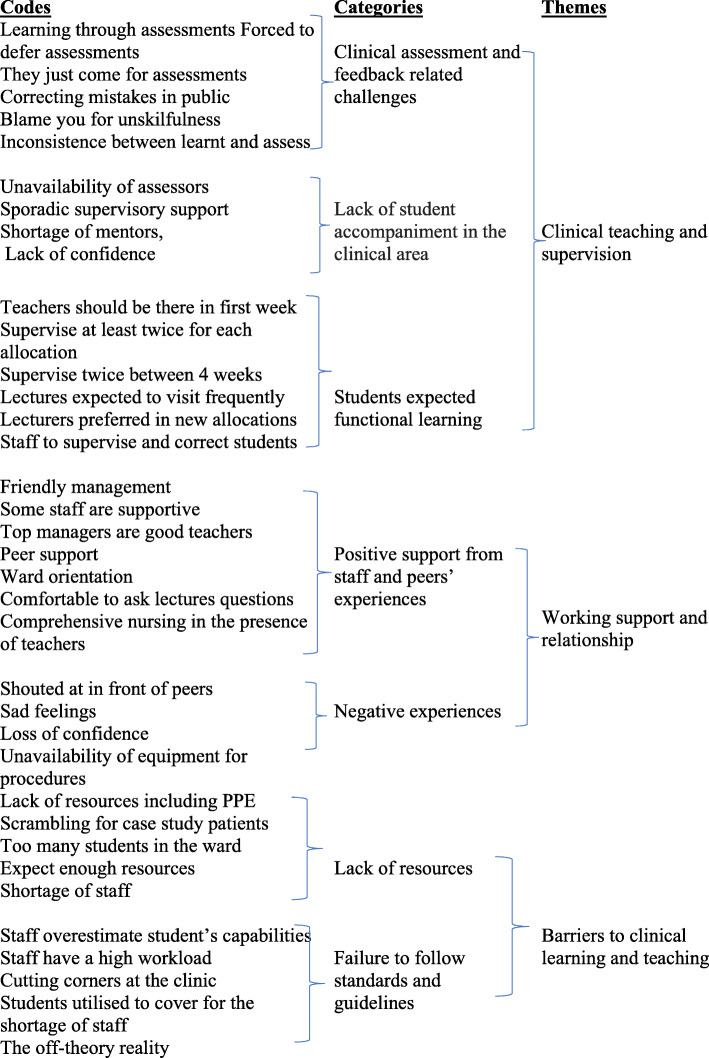
Qualitative data analysis and theme development

During the first phase of thematic analysis, AK and FL transcribed data and all other team members verified the transcription by re-reading the transcribed verbatim while listening to the recorded data. Areas that were incompletely or incorrectly transcribed were noted and corrected. Field notes were used to verify any atypical issues that arose during data collection and the interview environment. The second phase involved researchers who independently read and re-read the transcripts to identify patterns in the data. Authors MZ, AK and FL then developed a list of codes and collated the codes with extracts in a table. In the third phase of thematic analysis, the research team verified the codes and extracts before categorising the codes during their regular meetings. The groups of codes were then assessed further by the research team to identify potential themes in the fourth phase of thematic analysis. A coding tree was developed that had all the codes, categories and the identified themes (see Fig. 1). The inductive theme identification approach was used, as coding was based on participants’ experiences. In the fifth phase, the emerging themes were named and identified as main or subthemes by the research team. The research team reviewed all codes and themes emerging from the transcripts. Any discrepancies regarding data analysis were discussed among the researchers and agreement was reached through the majority rule. In the final phase, the researchers wrote a detailed account of the thematic analysis findings.

### Trustworthiness of data

The trustworthiness of data is assessed by credibility, dependability, conformability, transferability [29, 30]. Credibility entails trusting the findings of the study [30]. The research team ensured the credibility of the study findings in the following ways. Triangulation was done by comparing data from focus group discussions and surveys. The use of different methods compensates for limitations of individual methods at the same time maximising their strengths. Before the beginning of each focus group discussion, the participants were asked to provide honest responses. After transcription of the audio recordings, all participants were asked to review the transcripts. This provided a chance for the participants to highlight the areas that were missed or misunderstood. Dependability measures the extent to which similar results will be produced if the study was repeated in the same context, using the same population and methods. To ensure the dependability of the study findings, academics experienced in the qualitative study from Malawi and Norway, reviewed the methodology and examined the transcripts and the themes that emerged from thematic analysis. Confirmability describes the extent to which the researcher’s bias, motivation or interest are controlled, thereby ensuring that the findings of the study are determined by the respondents’ ideas and experiences and not the researchers’. This was achieved by taking field notes throughout the data collection process and triangulation of data sources. The field notes contained all important information such as methodological and logistic issues to ensure that important issues related to the research study were not missed out to aid in report writing. Finally, transferability, which assesses the applicability of findings, was achieved by a detailed description of steps undertaken from the study conception to reporting of the results to allow for study reproducibility.

## Results

### Survey

#### General characteristics of participants

Three-quarters of the participants were within the age group between 20 and 25 years old. The proportion of females was slightly higher than males (54% compared to 46%). More than three-quarters of the participants were nurse-midwife technician students pursuing a college diploma in Nursing and Midwifery. Most participants (57%) were in the third year of study (see Table 1 in Appendix 2).

The scores among the participants ranged from 97 to 164 (Mean [M] 131, standard deviation [SD] = 13.28). Satisfaction subscale had the highest mean score (M = 26.93, SD = 4.82), followed by personalisation (M = 23.27, SD = 4.02) while individualisation had the lowest mean score (M = 18.01, SD =3.50) (see Table 2 in Appendix 2). There was no significant difference between the total score of each subscale and age, gender, students study program and students’ training institution.

We used satisfaction subscale as the outcome measure, with the other subscales as explanatory variables. Students’ satisfaction with the clinical learning environment was positively correlated with all the other subscales. Pearson correlation coefficient ranged from 0.20 (Individualisation subscale, *p* = < 0.05) to 0.54 (Personalisation subscale, *p* = < 0.001) (see Table 3in Appendix 2).

Authors of research in the clinical learning environment using Fraser’s social-psychological conceptual framework [31] have found a relationship between satisfaction subscale scale and other CLEI subscales [32–34]. We, therefore, conducted multiple linear regression analysis to assess the association between satisfaction with clinical learning as a dependent variable and other psychosocial characteristics of CLEI (subscales) as independent variables. The findings of the multivariate analysis showed statistically significant association between satisfaction with clinical learning environment and personalization (β = 0.50, *p* = < 0.001) and task orientation (β =0.16 *p* = < 0.05). The two variables retained in the model explain 31% of the variability of the student satisfaction with their clinical learning environment (See Table 4 in Appendix 2).

#### Themes

Three focus groups, each per training institution, were conducted. Each focus group had 10 participants conveniently drawn from those who responded to the survey questionnaire. Three main themes emerged from the data, and these are 1) *Clinical teaching and supervision*; 2) *Working support and relationship,* and 3) *Barriers to clinical learning and teaching.*

### Clinical teaching and supervision

Participants shared their thoughts regarding clinical teaching and supervision. The discussions mainly focused on the following areas 1) *clinical assessment and feedback related challenges*, 2) *lack of student accompaniment in the clinical area,* and 3) *students expected functional learning.*

#### Clinical assessment and feedback related challenges

Students complained that clinical assessments were not done in time and that feedback was not always given to students. Some students further narrated how their clinical assessments for a particular departmental allocation were not undertaken throughout the clinical placement. For such students, an arrangement was made to defer and undertake the clinical assessments at a different hospital and at a convenient time to assessors. However, students complained that this strategy caused them to be assessed in different areas from what they had learnt in their original clinical placement. Others complained that deferring clinical assessments worked to students’ disadvantage because assessments piled up and caused stress as they were required to do multiple assessments within a short period. Below are the quotes from the students:

*“You find that most of the clinical assessments that were supposed to be done in the first year are carried forward to the third year, which puts pressure on us as we would have to do so many clinical assessments within a short period”* (Participant Focus group discussion [fdg] 2)

“*You will basically be at a new hospital that is totally different from the previous one, and they only come after the allocation to do assessments. You discover that what you have been learning is different from what you are being assessed on. You don’t even know they are coming for this carried forward assessment. I feel they should be assessing us on what we have been learning in that hospital allocation”* (Participant fdg 1)

Poor communication skills of the qualified nurses was another salient area discussed by participants. Students understood the benefits of being corrected by qualified nurses or lecturers during their practical placements; however, lack of privacy and confidentiality and criticism when correcting students robed them the dignity and confidence to try new skills. Others mentioned that they disliked being shouted at in front of peers. Examples of such narrations are as follows:

*“It happens that you are doing a procedure in the ward, and as a student, you are not as skilful and may fail to carry out the procedure competently. You find that staff members criticise you right there. This flattens my morale, and I don’t feel comfortable to do procedures with them [staff members]. I would love to be criticised in private when there is a problem and not in the presence of all patients because they lose trust in you”* (Participant fdg 1)

*“When a student is wrong, the qualified nurse would shout at you in the presence of patients and everyone”* (Participant fdg 3)

#### Lack of student accompaniment in the clinical area

Student discussed the unavailability of lecturers in the clinical area. The sporadic presence of lecturers at the clinical placement was attributed to many reasons, including the shortage of mentors. They further indicated that a lack of an adequate number of qualified staff in the clinical area also affected their clinical learning and experience. Many students reported that the wards had a shortage of staff, which resulted in inadequate supervision of students. This is narrated in the following quotes:

*“Previously, the clinical instructor used to accompany us to the hospitals. But these days, you find that you are allocated somewhere and you are left alone. They used to come with us; maybe it’s because of the transportation issues and the like”* (Participant fdg 3)

“*We have a shortage of human resource….we are failing to achieve our goals because of lack of mentors in the clinical area”* (Participant fdg 1)

#### Students expected functional learning

Students had expectations in the ward, which was not always the reality. Regarding assessments and availability of clinical instructors, students unanimously across the three focus groups reported that they wished their lecturers accompanied them to the clinical area. They valued the presence of clinical instructors in the first days of their allocation to assist with the familiarisation to the new environment. They further wished for the presence of qualified nurses, who were often busy to supervise, mentor and evaluate their daily clinical engagements as narrated below:

*“I think lecturers must be here [clinical area] at least for a week at the beginning of our allocation to orient us. Our current supervisory guidelines indicate that we should have at least not less than two visits from our supervisors. Let’s say we are doing labour and delivery for 4 weeks, coming twice within this allocation would be better than none”* (Participant fdg 1)

*“The qualified nurses should be able to supervise some of the procedures….so that when am doing something wrong at least they should tell me so that next time I should not do the same mistake”* (Participant fdg 3)

Students generally preferred lecturers because it was easier to approach them when confronted with a clinical problem and they were uncertain of the reactions of the health workers to students in the unfamiliar new allocations. Unlike when working under the supervision of nurses, students highlighted their comfort to ask questions and learn different skills if the clinical instructor or lecturer were present, which facilitated learning.

*“I expect that the lecturers should be visiting us frequently…. we are used to our lecturers and we feel free to ask them questions. In the ward, you are unsure of how the qualified staff will react to your question because you are new and unfamiliar”* (Participant fdg 2)

*“My expectation is that the lecturers should be visiting us frequently. If there is a condition in the ward, you are more open to asking the lecturers questions”* (Participant fdg 2)

### Working support and relationship

Discussions under this theme revolved around the *positive support from staff and peers’ experiences, and the negative experiences*.

#### Positive support from staff and peers’ experiences

Positive support was anything done to students that was perceived as helpful to their learning experience. Such positive support included a thorough hospital orientation by friendly management, ward orientation, peer education and support, and supportive staff. Positive feelings enhanced learning.

*“ if it is your first day at a specific ward, the qualified personnel or the head of a department welcomes you in a friendly manner and orients you around. You get comfortable because you now know the place”* (Participant fdg 3).

*“We are students from different schools; we meet in the clinical area, we learn through sharing what we learn in classrooms in our different colleges. So we try to assist one another”* (Participant fdg 3)

Some students reported top managers such as the District Health Officers (DHOs) and the District Medical Officers (DMOs) taught better about how to manage patients of different conditions. Notably, teaching support from senior managers was described as inspiring. Similarly, the presence of lecturers in the ward enhanced learning by encouraging students to provide comprehensive nursing care to patients. Below are the quotes from the students:

“*People like the DMO and DHO do have a heart for students and know what we need. They are knowledgeable about the practical management of conditions, and they can explain to you…sometimes they give us assignments to complete*” (Participant fdg 1)

*“When a lecturer is there, you do comprehensive procedures, like history taking will sort of be thorough and you learn through that”* (Participant fdg 2)

#### Negative experiences

Several students reported that their clinical experiences were negatively impacted by the poor relationship with some clinical staff. Students recalled some experiences when qualified clinical staff shouted at them in the presence of patients and fellow students. One student reported:

*“The qualified nurse shouted at me in the presence of patients and everyone. It spoiled my day, and I did not meet my objective that day because I was stressed up and annoyed….that wasn’t okay”* (Participant fdg 1)

### Barriers to clinical learning and teaching

Under this theme, barriers to acquiring skills and achieving their clinical objectives were discussed. Their discussion focused on the *lack of resources* and *failure to follow standards and guidelines.*

#### Lack of resources

Apart from human resources, students also mentioned a lack of resources to help them attain their clinical competencies, such as clinical equipment and protective gear. Lack of resources did not only fall below the students’ expectations but also facilitated poor quality of their clinical learning and the provision of substandard nursing practice through improvision of resources. Below are the quotes from the students:

*“When it comes to real practice, you find that most of the equipment or accessories that you learned in class are not available in the ward*” (Participant fdg 2)

*“Sometimes it is difficult because what you learn is from Western Countries and here in Malawi we do not have those resources, so we end up improvising, which is a challenge”* (Participant fdg 1)

Lack of patients was another reported resource-related barrier, and this happened as a result of an increased number of students in the ward from different institutions. Although a good relationship between students from these training institutions was generally reported, students were uncomfortable with having high numbers of counterparts from various institutions in the same ward. Having large numbers resulted in fighting over patients, as one student explained:

*“In the ward, you can have students from three nursing training institutions. All of you would want to identify case studies for assessments. We end up fighting over patients instead of assisting them”* (Participant fdg 3)

#### Failure to follow standards and guidelines

Students narrated that during the first weeks of their clinical placement, they tried to do what they learnt in class but over time, they also joined the qualified nurses in not following guidelines to perform procedures. The students reported that huge workload was the reason behind qualified nurses’ use of ‘shortcuts’ during procedures in the clinical area. Cutting corners was difficult for students to integrate theory into practice in the clinical area.

*“…..when you are with qualified nurses….. maybe it is due to high workloads, they cut corners and you learn nothing.”* (Participant fdg 1)

*“It becomes a problem to integrate what we learnt in class and what we meet in the ward. During classes, we learn the best way to conduct procedures, but when we go to the clinical areas, we sort of cut corners unless the clinical supervisor is around” (*Participant fdg 1)

While some students reported having support from the qualified nurses, others experienced an unwelcoming and unattractive learning environment, where ward staff expected them to do the work of a qualified nurse in the ward.

*“The qualified staff in the ward, most of the time think that if students are in the second, third or fourth year, they know everything, forgetting that we are not there to do their work but to learn….they just leave us to work unsupervised.”* (Participant fdg 3)

## Discussion

This study aimed to assess nursing and midwifery students’ experiences and perception of their clinical learning environment and to establish psychosocial characteristics of CLEI that are associated with satisfaction with the clinical learning environment. The results of the survey show that satisfaction followed by personalisation subscales had the highest mean scores, while innovation and individualisation had the lowest scores. Further, scores on satisfaction subscale were significantly higher in students who valued personalisation and task orientation. Concerning the qualitative findings, students reported that their clinical supervisors were unavailable to accompany or teach them in the clinical area. Assessments and feedback to students were also not conducted in time. Students also had difficulties in integrating theory into practice because of the lack of resources as well as qualified staff not following protocols when performing procedures, which affected them to achieve their clinical competencies. Students reported that they wished healthcare workers communicated to them properly, but that was not the case, as they were shouted at for not doing procedures properly.

This study has demonstrated that satisfaction with the clinical learning environment by the nursing students had the highest mean score. These findings are in agreement with results from a previous Australian study, where respondents demonstrated satisfaction with clinical placements [35]. Contrary to the findings of our study, authors of a Norwegian study found that personalisation sub-scale had the highest mean score [33]. The difference in findings between our study and the Norwegian study is likely to be related to limited opportunities among students to interact with clinical teachers in the clinical area given that the items in personalisation scale ask about clinical facilitators’ availability, interest in teaching and the support they provide to students during clinical practice. The qualitative findings of our study reveal that students received inadequate support from their clinical teachers, which may have influenced the overall score of the personalisation subscale to be slightly lower than that of satisfaction. In this study, students’ satisfaction with the clinical learning environment was positively correlated with all the other subscales. This demonstrates that satisfaction with clinical learning environment is dependent on multiple factors. Moreover, personalisation and task involvement were the main subscales, which contributed to satisfaction with the clinical learning environment in multiple linear regression. Evidence shows that students enjoy their clinical placement if they have opportunities to interact with the clinical instructor and have their concerns for their welfare considered in the clinical practice [32]. Having proper support in the clinical setting is essential for students considering that the Malawi Nursing and Midwifery Education Standards mandate students to spend 60% in the clinical setting and 40% in the classroom [36].

Although the results of the survey showed that the majority of students were satisfied with their clinical learning environment, most students in the focus groups were dissatisfied with the level of support in clinical teaching and supervision. They cited a lack of proper guidance and continuous supervision by lecturers and qualified members of staff. This divergent finding could be explained by the differences in the two methodologies used in this study. In the qualitative study, the participants were given the freedom to explain and had an in-depth discussion, unlike in the survey where the CLEI tool restricted participants to describe their feelings. This finding is similar to that of a mixed-method study conducted with nursing students in Australia where students reported lower levels of satisfaction with the clinical learning environment in quantitative findings but this was not supported by the qualitative findings [37]. Lack of support in clinical teaching and supervision affects students’ learning experience in the clinical setting because students value familiarity, acceptance, trust, support, respect and recognition of their contribution to patient care in the clinical area [38]. Support from the lecturers and tutors during clinical practice helps to allay fears and anxieties, provides guidance and encouragement to acquire the requisite knowledge, skills and attitudes for practice, which in turn helps the students to provide high-quality patient care. During the first clinical placement, students are very anxious due to unfamiliarity of caring for patients and fear of making mistakes.

Additionally, the study results have also demonstrated the challenges that students face in integrating theory into practice due to inadequate support from the lecturers, lack of resources and failure of qualified members of staff to provide comprehensive care to patients. Conflicting practices between the ideal nursing taught in the classroom and that of the clinical setting result in students being confused, stressed and anxious if they are not well taught and supervised [39]. This, therefore, has implication for the academic institutions and teaching hospitals in Malawi to identify and come up with better means of supporting students in the ward. The nursing training institutions should consider allocating more clinical supervisory hours for lecturers. At the same time, the hospitals should promote professional integrity in qualified nurses to provide standard nursing care in alignment with institutional policies and guidelines and play as role models to students.

The results of our study also show that students were not happy with how the clinical assessments and feedback from the lecturers and qualified staff were conducted during clinical practice. Learning during clinical placement takes place if students understand the right and wrong actions. The clinical nurse educator’s role is to enhance learning through the provision of learning opportunities, supporting, guiding and conducting fair and timely evaluations. This builds on the findings from a study conducted in Iran where nursing students felt unsatisfied with their clinical assessments and evaluations because they were done by nursing staff who they believed lacked knowledge and experience in assessments and feedback [9]. Feedback helps students to gain confidence by reinforcing good performance and highlighting areas needing improvement [40]. Several studies have illustrated measures to try and close the theory-practice gap through reflection and problem based learning under the guidance and support of lecturers and clinical staff that help them to develop their critical thinking and problem-solving skills in clinical practice. Students, therefore, need to be adequately taught, supervised and encouraged to link theory learnt in class with the realities of nursing practice [41].

Results of this study also revealed that students experienced a negative working relationship with clinical staff. These results are consistent with those reported in a study conducted in Greece, where students reported that qualified nurses were hostile and communicated poorly to students [32]. Good interpersonal relationship, communication and support between staff and students create a conducive environment which is essential for student learning in the clinical setting. Such behaviours reduce anxiety and foster socialisation process, confidence and self-esteem, thus promoting clinical learning [42].

Some students reported that they were doing routine tasks and sometimes non-nursing duties while others reported a variety of learning opportunities which facilitated their learning. These learning opportunities were compromised by workload and overcrowding of students. Porter and colleagues [43] suggested that students have to be given opportunities to practice different tasks to gain confidence, become perfect and learn from the mistakes. While this suggestion is ideal, the number of students in nursing colleges has increased such that the students are not given adequate opportunities to learn. The overcrowding of students in the clinical setting affects peer support which could lead to conflicts, tension, competitions for opportunities and lack of fulfilment of some requisite competencies, which in turn compromise the care given to patients during clinical practice [44]. Teaching hospitals and nursing training institutions should work together and devise plans and strategies that can allow a reasonable number of students to undertake their clinical practice at a specific period. This strategy would not only reduce congestion of students in the hospital but also provide more opportunities for skills development through comprehensive learning from both qualified nurses and their lecturers.

The study results revealed that both human and material resources were inadequate for the clinical learning experience of nursing and midwifery students. Teaching and learning resources are critical in nursing and midwifery education. To provide high-quality nursing care to patients, student nurses need to learn theoretical knowledge as well as practical skills. Lack of both time and material resources to facilitate learning can lead to students feeling unsupported. Literature suggests that nurse educators are expected to accompany student nurses to the clinical area. However, this is often not possible in Malawi due to the shortage of academic staff in nursing training institutions [45]. Lack of guidance and supervision may lead to nursing students learning incorrect procedures, become incompetent and lose interest in the nursing profession as they feel frustrated [46].

Donough and Van der Heever [15] state that professional nurses are responsible for teaching, supervising, guiding, counselling, assessing and evaluating student nurses in the clinical area. The results of this study revealed that professional nurses in the clinical setting were busy with their administrative roles and patient care but were less supportive of students. Similar results were reported by authors of a study in Taiwan, where staff shortages caused patient care to take priority over clinical teaching of student nurses [47]. Nursing and midwifery training institutions in partnership with the clinical practice facilities are responsible for preparing student nurse-midwives to cope with the complexity and nature of clinical practice by ensuring that both human and material resources are available and adequate to enhance students’ clinical learning [48].

### Limitations

This study was conducted in three training institutions in Northern Malawi. As such, it may not be a representative of the experiences of all the nursing and midwifery students in Malawi. Also, the small sample size in this study may affect the generalizability of the findings. Our study only used the ‘Actual Form’ of CLEI to assess students’ perception of the real clinical learning environment, and not the ‘Preferred form’ to assess their perception of the characteristics of the desired clinical learning environment. This may be considered as one of the limitations of our study. A comparison of students’ perceived and preferred clinical learning environment would have complemented the explanation of the divergence of our mixed study finding (CLEI vs focus group). Therefore, the findings of the study should be interpreted with caution. Limitations of our study propose the need for conducting a larger study, using both ‘Actual Form and Preferred Form’ of CLEI that can be generalised and give a more substantial direction.

## Conclusion

The findings of this study show that although students described their clinical learning environment as satisfactory using CLEI, findings from the focus group discussions revealed that students had many challenges that impacted their clinical learning. Hostile environment, poor relationship with the qualified nurses, absence of lecturers and lack of resources were some of the factors that affected students’ clinical learning experience. The findings of this study underscore the need to improve all aspects of the learning environment. For example, training institutions need to have a clear clinical supervision plan for students to ensure that a clinical supervisor is available to teach and assess students. Qualified staff also need training on how they can better support students in the clinical area despite their high workload. Hospitals and training institutions also need to plan for the availability of essential equipment in the clinical area to help students gain the required clinical skills.

## Data Availability

The data and all supporting materials used in our manuscript are freely available to any scientist wishing to use them from the corresponding author on request.

## Appendix 1

### Interview guide for focus group discussions with students

1. Describe the clinical learning experiences in the recent clinical setting that you have been?
2. What is your experience in integrating theory to practice during that clinical placement?
3. What are your expectations of learning in the clinical placements-

- from teachers
- from nurses
- from environment
- from clinical management

4. Describe the teaching approaches used during your clinical placement.
5. Describe the working relationship in the clinical learning environment.
6. Explain how the clinical environment is effectively meeting your objectives.
7. What challenges (if any) do you experience in the clinical placement?

## Appendix 2

### Results

**Table 1:** General characteristics of the study participants (*N* = 126)

Variable	n (%)
**Age**	
≤ 20	4 (3)
20–25	96 (76)
≥ 25	26 (21)
**Sex**	
Female	68 (54)
Male	58 (46)
**Program of study**	
BSc Nursing & Midwifery	15 (12)
Nurse Midwife Technician	111 (88)
**Year of study**	
Second	39 (31)
Third	72 (57)
Fourth	14 (11)
Missing	1 (1)
**Clinical placement**	
Central hospital	44 (35)
District hospital	54 (43)
Others	25 (20)
Missing	3 (2)
**Recent clinical Placement***	
Medical	10 (8)
Surgical	13 (10)
Labour and delivery	29 (23)
Postnatal	10 (8)
Antenatal	11 (9)
Paediatric	32 (25)
Family planning	3 (2)
Under 5 clinic	2 (2)
Theatre	7(6)
Missing	9 (7)
**Length of current clinical placement**	
< 3 weeks	14 (11)
3-8 weeks	77 (61)
> 8 weeks	31 (25)
Missing	4 (3)

**Table 2:** Mean scores of total and subscales of CLEI Actual form (*N* = 126)

CLEI Scale	Mean ± SD	Range	Minimum	Maximum
CLEI total scale	131.29 ± 13.28	67	97	164
Satisfaction	26.93 ± 4.82	25	9	34
Personalization	23.27 ± 4.02	20	12	32
Student involvement	22.67 ± 3.04	22	12	34
Task orientation	21.73 ± 3.52	18	13	31
Innovation	18.68 ± 2.89	16	12	28
Individualization	18.01 ± 3.50	20	7	27

**Table 3:** Correlation between satisfaction subscale and other subscales of the CLEI Actual form

CLEI Subscales	R	*p*-value
Personalization	0.54	0.000
Student involvement	0.23	0.005
Task orientation	0.30	0.000
Innovation	0.27	0.001
Individualization	0.20	0.014

**Table 4:** Multiple linear regression with satisfaction as a dependent variable and other subscales of the Actual CLEI scale as independent variables

Independent variables	Beta (95% confidence interval)	*p*-value	R^2^	F
Personalization	0.50 (0.41–0.78)	0.000	0.31	28.35
Task orientation	0.16 (0.12–0.43)	0.038		

## Abbreviations

ANOVA: Analysis of Variance
CLEI: Clinical Learning Environment Inventory
M: Mean
NHSRC: National Health Research Committee
SPSS: Statistical Package for the Social Sciences
SD: Standard Deviation
WHO: World Health Organisation
